# Diagnostic performance of Pneumonia multiplex PCR in critically ill immunocompromised patients

**DOI:** 10.1186/s13054-025-05528-y

**Published:** 2025-07-17

**Authors:** Jérémy Contier, Laura Platon, Nacim Benchabane, Sonia Tchakerian, Fanchon Herman, Caroline Mollevi, Patrice Ceballos, Sylvain Godreuil, Kada Klouche

**Affiliations:** 1 Department of intensive care medicine, Lapeyronie University Hospital, University of Montpellier, Montpellier, France; 2 Department of statistics, Lapeyronie University Hospital, University of Montpellier, Montpellier, France; 3 Department of Hematology, Saint Eloi University Hospital, University of Montpellier, Montpellier, France; 4 Department of Microbiology, Lapeyronie University Hospital, University of Montpellier, Montpellier, France; 5 PhyMedExp, School of Medicine, INSERM (French Institute of Health and Medical Research), CNRS (French National Centre for Scientific Research), University of Montpellier, Montpellier, France

**Keywords:** Antibiotic, Immunocompromised, Pneumonia, Multiplex polymerase chain reaction, Acute respiratory failure, Intensive care unit

## Abstract

**Background:**

Admissions of immunocompromised patients to intensive care units (ICUs) are on the increase. The main reason for admission is acute respiratory failure, predominantly of infectious origin. In such circumstances, early and appropriate antibiotic therapy guarantees a better prognosis. Rapid diagnostic techniques such as multiplex polymerase chain reaction (PCR) have shown their value in both diagnosis and treatment in immunocompetent patients. To date, little data are available on immunocompromised patients.

**Methods:**

In this retrospective, single-center study, we analyzed data from critically ill immunocompromised patients admitted for acute respiratory failure requiring invasive ventilation, in whom a respiratory specimen was taken and processed simultaneously by BioFire FilmArray Pneumonia Panel multiplex PCR (BFPPm PCR) and conventional culture (CC). Samples had to be taken from deep respiratory tracts less than 48 h after mechanical ventilation. The primary endpoint was the evaluation of the diagnostic performance of BFPP mPCR compared with CC. The secondary endpoint was the therapeutic impact of the results of BFPP mPCR.

**Results:**

One hundred and fourteen patients were included, with immunosuppression mainly of a hematological (35.1%) and oncological (35.1%) nature. The mPCR positivity rate was 36.8%, with the majority identifying enterobacteria (51%) and a median turnaround time of between 2h30 and 4 h. Comparison of rapid techniques with CC showed sensitivity of 89%, specificity of 83%, predictive positive value of 52% and negative predictive value of 98%. Concordance between the two techniques was complete in 84.2% of cases. mPCR enabled antibiotic therapy to be modified in 17.5% of cases, mainly de-escalation.

**Conclusion:**

The use of mPCR in the diagnosis of pneumonia in immunocompromised patients shortens the time required to obtain results, and is particularly effective in eliminating the presence of multi-resistant germs. Bacteria detected in culture and not included in the mPCR spectrum were mostly bacteria of low pathogenicity or sensitive to the antibiotics usually prescribed. The mPCR technique could reduce exposure to broad-spectrum antibiotics in this population.

**Supplementary Information:**

The online version contains supplementary material available at 10.1186/s13054-025-05528-y.

## Introduction

Recent years have been marked by improved management of onco-hematology patients and the widespread use of immunosuppressants. As a consequence, there is a relentless rise in the number of admissions of these immunocompromised patients to intensive care units (ICUs) [1–3]. However, their mortality remains high, particularly when mechanical ventilation is required [4, 5]. Indeed, acute respiratory failure (ARF) remains the leading reason for admission to the ICU, and is most often of infectious origin. Pathogens include community and non-community acquired bacteria, viruses and fungi [6]. In view of the clinical severity of the infection, probabilistic antibiotic therapy is generally initiated as a matter of emergency. At the same time, the causative organism is identified using non-invasive and/or invasive tests and conventional methods. However, this usually takes 48 to 72 h, and is only cost-effective in about half of all cases [7]. Initial antibiotic therapy is then readapted according to the results of positive cultures. In fact, this antibiotic therapy is sometimes inappropriate, either being too broad or too narrow spectrum. In case of the former, the patient’s flora is subjected to a selection pressure of resistant mutants, and there is an increase in mortality with over-treatment [8–10]. In the later case, the infection is not controlled, as it is linked to an unusual agent not covered by the initial probabilistic antibiotic therapy. To be in line with the antibiotic stewardship policy recommended in recent years, which consists of the measured use of broad-spectrum antibiotics, with de-escalation to the narrowest possible spectrum, we need to have access to faster, more reliable microbiological diagnostic techniques [11].

Recently, rapid diagnostic tools have been developed, the most interesting of which are those using polymerase chain reaction (PCR). The FilmArray^®^ Pneumonia Panel plus test is based on the detection of deoxyribonucleic acid (DNA) from pathogens involved in infection. Available PCR primers detect 18 bacterial frequently implicated in pneumonia. They can also detect 9 different genetic markers of antibiotic resistance. The DNA purification method (BioFire technology) enables the test to be performed from a variety of culture media and samples like: sputum, tracheal aspirates or bronchoalveolar lavage (BAL) fluid. Results can be obtained within 1 to 3 h.

In our center, since March 2020, we have been routinely using molecular diagnostic methods on respiratory samples from immunocompromised hosts requiring an invasive mechanical ventilation. Though BioFire^®^ FilmArray^®^ Pneumonia Panel (BFPP) mPCR has been shown to have a good diagnostic performance in immunocompetent critically ill patients, little data exist in immunocompromised patients [12]. We then conducted this retrospective study to compare the diagnostic performance of BFPP mPCR on our immunocompromised patients to that of conventional cultures (CC). We also sought to investigate the potential impact of BFPP mPCR on antibiotic management in these patients.

## Materials and methods

### Population

All consecutive immunocompromised patients, ≥ 18 years of age, admitted to Lapeyronie Montpellier University Hospital ICU, between March, 2020 and March 2023, for hypoxemic ARF requiring mechanical ventilation were included in the study. We selected patients suspected of having pneumonia, in whom a microbiological sample was taken within the first 48 h after orotracheal intubation, and whose bacteriological analysis included CC and BFPP mPCR. Patients with ventilation-associated pneumonia (VAP), occurring more than 48 h after initiation of invasive mechanical ventilation or occurring during ICU stay were excluded.

Immunodepression was defined according to the following criteria: cancer < 5 years, hematologic malignancy, hematopoietic stem cell transplant, solid organ transplant or autoimmune disease under immunosuppressive therapy. Pneumonia was suspected if an inflammatory syndrome and/or fever were associated with a radiographic (opacity or alveolar-interstitial syndrome) and/or CT scan image (alveolar condensation or ground-glass opacity).

The following data were collected for each patient: age, sex, type of immunosuppression, characteristics on ICU admission, including SOFA and SAPSII scores, diagnostic features of pneumonia, organ failures during ICU stay and eventual resolution of pneumonia.

### Microbiological sampling conditions

A respiratory sample, either from a tracheal aspirate, a mini-BAL or a BAL, was taken from each patient. The sample was microbiologically analyzed in the conventional way, with direct examination, culture and antibiotic susceptibility testing if a germ was identified. At the same time, a Biomérieux^®^ BFPP mPCR was performed on this sample.

The BFPP mPCR detects 18 bacteria, including 3 atypical bacteria and 7 antibiotic resistance genes (Supplementary Table 1). Some bacteria such as *Citrobacter spp*, *Hafnia alvei*, *Morganella morganii*, *Stenotrophomonas maltophilia* or *anaerobes* are not detectable.

Bacteria detected by each technique were collected. Culture and/or PCR were considered positive when the presence of bacteria was detected by either technique.

### Outcome

The primary outcome was the diagnostic performance of the mPCR panel as compared to CC, considered as gold standard. Only germs included in the mPCR panel were included in this analysis. The PCR result was classified as true positive (TP) if both techniques were positive for both the same bacterium and bacterial count, and as true negative (TN) if both techniques were negative. The PCR result was classified as false negative (FN) if at least one mismatch was observed, i.e. a germ (included in the mPCR panel) detected by CC was not detected by BFPP mPCR. It was classified as a false positive (FP) if the germ was detected by the BFPP mPCR and not by the CC. If at least two mismatches were observed, i.e. a germ detected by CC and not by PCR, and a germ detected by BFPP mPCR and not by CC, the PCR result was classified as FP.

The secondary objective was to study the impact of BFPP mPCR and CC data on antibiotic therapy management. De-escalation and escalation of antibiotic therapy was judged according to the spectrum of antibiotics used. The levels are shown in ascending order of spectrum in supplementary Table 3. Antibiotic de-escalation was defined as the discontinuation of one of the current antibiotics or a reduction in the spectrum of current antibiotic therapy (supplementary Table 2). Discontinuation of the anti-staphylococcal antibiotic is considered de-escalation. Antibiotic escalation was defined as the addition of a new antibiotic or broadening the spectrum of current antibiotic therapy. Adaptation was defined as the modification of current antibiotic therapy without reduction or broadening of the spectrum.

### Statistical analysis

Qualitative variables were reported as numbers and percentages, and quantitative variables as means with standard deviations, or as medians with 25th and 75th percentiles.

The primary endpoint was the diagnostic accuracy of BFPP mPCR compared with CC in terms of sensitivity (Se), specificity (Sp), positive predictive value (PPV) and negative predictive value (NPV):

- Se is calculated in subjects with a positive reference test (CC): Se = TP/(TP + FN).
- Sp is calculated in subjects with a negative reference test (CC): Sp = TN/(TN + FP).
- PPV is calculated in subjects with a positive index test (mPCR): PPV = TP/(TP + FP).
- NPV is calculated in subjects with a negative index test (mPCR): NPV = TN/(TN + FN).
- Se, Sp, PPV and NPV were calculated and presented with their 95% confidence intervals.

All statistical analyses were performed using R statistical software, version 3.3.2 (http://www.R-project.org, Free Software Foundation, Boston, USA).

## Results

### Study population

**Fig. 1:**
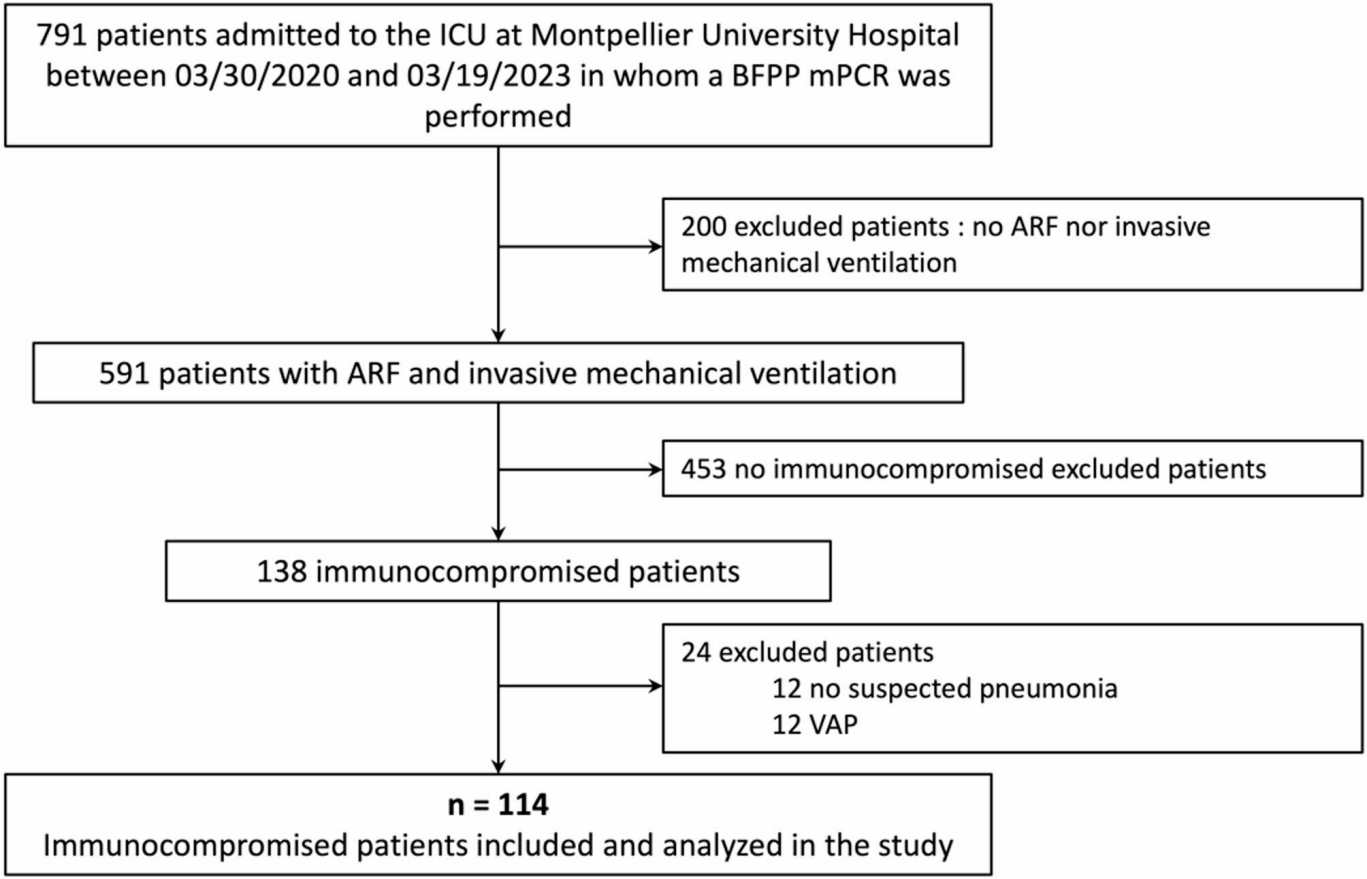
Flow chart of the study population. *ARF: acute respiratory failure; BFPP: BioFire® FilmArray® Pneumonia Panel; mPCR: multiplex polymerase chain reaction; ICU: intensive care unit; VAP: ventilation-acquired pneumonia*

During the study period, 591 patients with ARF requiring invasive mechanical ventilation were admitted to the ICU. Of these, 138 patients were immunocompromised and eligible for inclusion. After exclusion of 24 patients who did not meet our criteria after review of the medical record, 114 patients were included and analyzed (Fig. 1).

The population was predominantly male, with a median age of 65 yo, a median SAPSII of 53 and a SOFA score of > 8 in more than half the patients (Table 1). Immunosuppression was mainly due to onco-hematological disease. Nearly 9% of patients were multi-resistant bacterial carriers on admission. None of the patients had received antibiotic therapy in the 30 days prior to ICU admission, with the exception of antifungal prophylaxis (pneumocystis j. and aspergillus.) when indicated.

**Table 1:** Characteristics of the study population

Variable	*n*	Population
Demographics data		
Age in years, median (25%; 75%)	114	65.5 (57.2; 70.3)
Male, n (%)	114	74 (64.9)
Type disease with immunodepression, n (%)	114	
* Hematology*		40 (35.1)
* Oncology*		40 (35.1)
* Solid organ transplantation* * Immunosuppressive therapy*		20 (17.5) 14 (12.3)
Admission to ICU		
Invasive ventilation, n (%)	114	24 (21.1)
Hemodynamic failure, n (%)	114	43 (37.7)
Aplasia, n (%)	114	15 (13.2)
SAPS II, median (25%; 75%)	114	53.0 (42.0; 71.5)
SOFA, median (25%; 75%)	114	8.5 (6.0; 11.0)
Diagnostic of pneumonia		
Fever, n (%)	103	48 (46.6)
Acute respiratory distress, n (%)	105	78 (74.3)
Radiographic sign, n (%)	113	103 (91.2)
Scanographic sign, n (%)	99	95 (96.0)
CRP in mg per liter, median (25%; 75%)	114	116.0 (60.0; 208.8)
During ICU stay		
Duration of invasive ventilation in days, mean ± SD	114	11.7 ± 13.3
Worst PaO2/FiO2 ratio, n (%)	113	
* [100; 200]*		37 (32.7)
* [200; 300]*		13 (11.6)
* < 100*		63 (55.8)
Use of vasopressor support, n (%)	114	107 (93.9)
Use of renal replacement therapy, n (%)	114	41 (36.0)
Length of stay in days, median (25%; 75%)	114	12.0 (7.7; 17.9)
Resolution of pneumopathy, n (%)	114	71 (62.3)
ICU mortality, n (%)	114	45 (39.5)

*CRP: C reactive protein; SAPS II: Simplified Acute Physiology Score II; SOFA score: Sepsis-related Organ Failure Assessment score; ICU: intensive care unit*

### Samples and pathogen identification

Respiratory samples were mostly from BAL (74.6%), and the median time from ICU admission to completion was 1.2 days (Supplementary Table 3).

**Fig. 2:**
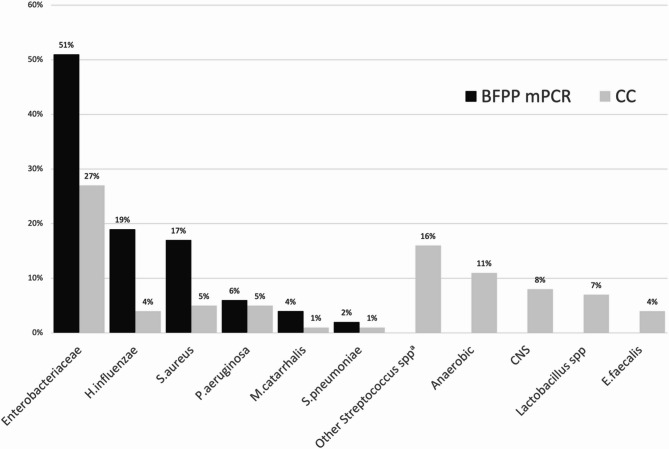
Incidence of bacteria detected by each of the 2 techniques BFPP mPCR: BioFire® FilmArray® Pneumonia Panel; CC: conventional culture; CNS: coagulase-negative *Staphyloccocus*; PCR: polymerase chain reaction^a^*Streptococcus*except*S.pneumoniae, S.agalactiae and S.pyogenes*

Bacteriological results were as follows: 29.8% of samples were positive by mPCR and 40.4% by CC. Organisms identified by mPCR were enterobacteria in 51% of cases, with a majority of *E.coli* (28%), followed by *K.pneumoniae* (13%) and *Enterobacter spp* (6%). Other germs identified were *H. influenzae* (19%), *S. aureus* (17%). *P. aeruginosa* accounted for only 6.4% of germs identified by PCR (Fig. 2).

Germs identified by CC were also mainly *Enterobacteriaceae* (27%), with a majority of *E.coli* (13%), followed by *K. pneumoniae* (6%) and *Enterobacter spp* (2%).

In a significant number of cases, bacteria identified by CC were not detectable by PCR. These included *streptococci*, excluding *S.pyogenes*,* S.agalactiae* and *S.pneumoniae*, in 16.5% of cases, anaerobic germs in 10.5% of cases, coagulase-negative *Staphylococcus* in 8.2% of cases, and *Lactobacillus* in 7.1% of cases. On the other hand, CC identified 4.7% of samples as positive for *S.aureus*, 4.7% for *P.aeruginosa*, and 3.5% for *H. influenza*, a lower positivity rate than PCR, as well as for *S.pneumoniae* (only one culture-positive sample) (Fig. 2). Only one case of multi-resistant bacteria was observed: 1 extended-spectrum beta-lactamase (ESBL)-producing enterobacteria detected by both PCR and CC. No cases of methicillin-resistant *Staphylococcus aureus* (MRSA) or carbapenemase were found.

Of note, a significant number of mycological samples were positive. Aspergillus antigen was found in 20% of respiratory samples and 5% of blood samples. Cultures were positive for Aspergillus spp in 10% of respiratory samples. PCR identified Pneumocystis J in 13% of respiratory samples. Antifungal therapy was started as soon as the results were received.

### Diagnostic performance

After exclusion of culture-positive isolates undetectable by mPCR, complete concordance of PCR with CC was 84.2%, and partial concordance in 89.5% of cases.

**Fig. 3:**
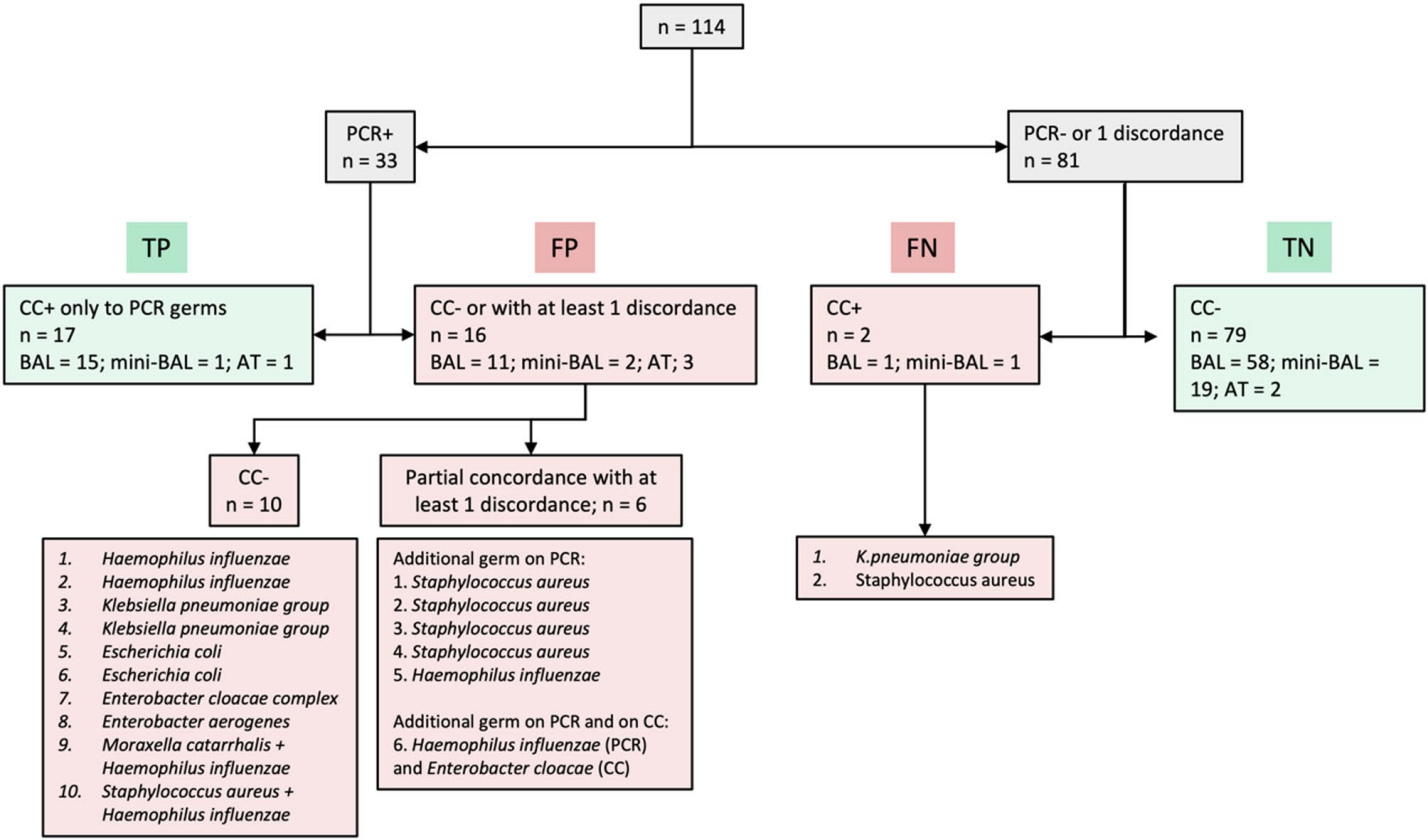
PCR and conventional culture results for BFPP panel bacteria. *PCR: polymerase chain reaction; CC: conventional culture; TP: true positive; FP: false positive; FN: false negative; TN: true positive*

A positive BFPP mPCR for at least one bacterium was found in 33 samples. In 17 cases, the same bacterium was identified by both techniques (TP), and in 79 cases, no bacterium was detected by either technique (TN). However, 10 samples were PCR-positive but CC-negative (FP), and a further 6 samples were PCR-positive with at least one additional bacterium not detected by CC (FP). In over a third of these cases, the bacterium in question turned out to be *H. influenzae*, and in a further 31%, *S.aureus*. Of these 16 samples, 13 (81.3%) were taken by BAL more than 24 h after initiation of antibiotic therapy. Finally, one germ was identified by CC but not by PCR (FN): a *K. pneumoniae *(Fig. 3). These diagnostic performances are summarized in Table 2.

Taking into account samples where both PCR and CC were positive for at least one germ, defining partial or complete concordance, the Se was 96.0% [79.7; 99.9], the Sp 88.8% [80.3; 94.5], the PPV 70.6% [52.5; 84.9] and the NPV 98.8% [93.2; 100.0].

Considering all germs, almost 2/3 of bacteria detected by CC were not detectable by BFPP mPCR. These were: commensal Streptococci (16%), coagulase-negative *Staphylococci* (8%), *Enterococcus faecalis* (4%), *anaerobes* (12%), and *Enterobacteriaceae* such as *M.morganii* (1%) and *Citrobacter spp* (2%).

**Table 2:** Contingency table: biofire^®^filmarray^®^pneumonia panel multiplex polymerase chain reactive diagnostic performance compared to conventional culture

	**CC +**	**CC -**	**Total**
BFPP mPCR +	17	16	33
BFPP mPCR -	2	79	81
Total	19	95	114

BFPP mPCR	Estimation	CI95%	
Sensitivity	0.89	[0.67; 0.99]	
Specificity	0.83	[0.74; 0.90]	
PPV	0.52	[0.34; 0.70]	
NPV	0.98	[0.91; 1]	

### Antibiotic therapy

Empirical antibiotic therapy was started 1.2 ± 1.9 days before sampling. Initial antibiotic therapies mostly combined Piperacillin-Tazobactam or penems or broader-spectrum antibiotics (see supplementary Table 3, levels 7 and 8) with a major antistaphylococcal agent (linezolid or vancomycin). A single dose of aminoglycoside (amikacin) has been added in cases of septic shock. Any occurrence of a documented infection or institution of antibiotic therapy during 6 months before ICU admission was taken into account and used as a guide for the choice of empirical antibiotic therapy. In this case, the spectrum of initial empirical antibiotic therapy was broadened. No antifungal agents were used empirically.

Antibiotic therapy was modified in 20 patients (17.5%) after receipt of mPCR results, and consisted of antibiotic de-escalation, with a narrowing of the spectrum in more than half of these (11 patients) (Table 3). This modification was appropriate in 89.5% of cases after retrospective reading of culture results. The antibiotics most frequently spared were the penems.

The median delay for mPCR was between 2 h and 30 min and 4 h in our hospital.

**Table 3:** Impact of the results of each technique on antibiotic stewardship

Variable	*n*	Population
Antibiotic therapy modified with BFPP mPCR result, n (%)	20	20 (17.5)
* Adaptation*		2 (10.0)
* Stop*		3 (15.0)
* Desescalation*		11 (55.0)
* Escalation*		2 (10.0)
* Introduction*		2 (10.0)
* Modification considered suitable a posteriori*,* n (%)*		17 (89.5) ^a^
Antibiotic therapy modified with CC result, n (%)	25	25 (21.9)
* Adaptation*		4 (16.0)
* Stop*		3 (12.0)
* Desescalation*		15 (60.0)
* Escalation*		2 (8.0)
* Introduction*		1 (4.0)

*CC: conventional culture; BFPP mPCR: BioFire® FilmArray® Pneumonia Panelmultiplex polymerase chain reaction*^a^ 17/19 patients because on one missed data

## Discussion

In this cohort of critically ill immunocompromised patients, the diagnostic performance of BFPP mPCR was assessed against reference CC. We found that BFPP mPCR had satisfactory diagnostic performance with complete agreement with CC in 84.2% of cases, and partial agreement in 89.5% of cases. Interestingly, BFPP mPCR had an excellent NPV, allowing us to exclude infection by a panel bacterium. When we received the mPCR results from BFPP, we were able to modify the antibiotic therapy early in almost one case out of five, mainly by de-escalating the antibiotic. BFPP mPCR also guaranteed rapid results, less than 4 h after sampling.

Several authors have reported the usefulness and benefit of BFPP mPCR in critically ill patients [12]. However, data on critically ill immunocompromised patients remain scarce. We therefore compared the diagnostic performance of BFPP mPCR with that of CC in critically ill immunocompromised patients. Our population is representative of a critical population of immunosuppressed patients, given the causes of immunosuppression, severity scores and mortality rates observed [13]. Of the 114 samples, 33 (29.8%) identified a bacterium by BFPP mPCR, more than half of which were enterobacteria, while the remaining 81 were negative. The search for resistant germs using this technique was positive only once, when a single ESBL-producing enterobacterium was detected. In comparison, more samples (46/114, 40.4%) were positive by CC, including samples positive for bacteria not detected by PCR as part of the so-called PCR gap (anaerobes, coagulase-negative *Staphylococcus* (CNS), *Lactobacillus*, other *Streptococcus spp*.). Nevertheless, most of these bacteria identified by CC and not by PCR were of low virulence, and their pathogenicity remained questionable, especially when they were part of a polymicrobial pneumopathy such as CNS, *Lactobacillus spp*, and *Streptococcus spp.*, are generally susceptible to conventional antibiotic treatment. In contrast, we found that a significant number of mPCR-positive samples identifying *H.influenzae* were not confirmed by CC. Indeed, only a third of *H.influenzae*-positive BFPP mPCR were confirmed by culture. This can be explained by the well-known difficulty of growing and the need for a specific culture medium for this bacterium. Whether the molecular diagnostic method too sensitive, or whether it can identify bacteria that escape CC, is a matter for debate. However, the shortcomings of PCR, particularly for Citrobacter spp, Hafnia alvei, Morganella morganii, Stenotrophomonas matophilia and anaerobes, must be taken into account when interpreting PCR. Indeed, cases involving strict anaerobes or other Enterobacteriaceae spp, would be more problematic and warrant adaptation of antibiotic therapy.

By excluding bacteria not included in the BFPP mPCR panel, the concordance of this technique with the conventional technique proved satisfactory, with a Se of 89% and a Sp of 83%. Moreover, its NPV was high, close to 100%. This means that a negative mPCR result would enable us to safely exclude the main bacteria frequently observed in such a population, such as *P.aeruginosa*, ESBL or MRSA, which would justify broad-spectrum antibiotic therapy. Conversely, the PPV was low: only half of PCR-positive samples were CC-positive. BFPP mPCR may have a much higher Se than CC, detecting bacteria dismantled by an antibiotic introduced prior to sampling. On the contrary, it could be too sensitive, leading to over-diagnosis of sometimes non-pathogenic bacteria, by detecting the DNA of non-viable bacteria or satellites of simple colonization.

Diagnostic performance of mPCR has been evaluated previously but mainly in immunocompetent patients. A retrospective multicentric study, including both immunocompetent and immunocompromised patients, reported a good diagnostic performance of mPCR in pneumonia with a Se of 85,7%, a Sp of 98,4% and NPV of 97,9% [14]. *Sircar et al.* [15] compared, in a retrospective case-control study, the concordance of mPCR and CC results on BAL samples. Concordance between the two techniques was complete in 55.2% of cases, with the majority of discordant cases being a positive mPCR associated with a negative CC. In the multicenter diagnostic study by *Murphy et al.* [12], including immunocompetent patients, BFPP mPCR had a Se of 75 to 100%, and a Sp of 87.2 to 100%, depending on the bacteria. Data for immunocompromised patients are indeed limited. A multicentric trial on patients with hematologic malignancy showed that detection of viruses on mPCR nasopharyngeal in ICU is associated with mortality [2]. Also, a retrospective trial in immunocompromised patients performed in India, found that mPCR was positive in 59% of samples whereas CC in 37% only. The panel used in this trial was larger than BFPP, including *cytomegalovirus*, *P.jirovecii*, *Aspergillus* spp and *Bocavirus* [16]. *Azadeh et al.* [17] compared BAL mPCR with nasopharyngeal mPCR in immunocompromised patients and found a concordance between the two sites at 89%; but they did not evaluate concordance with CC. Lastly, a recent retrospective study involving 24 patients has compared mPCR with CC, specifically in immunocompromised patients (solid organ transplantation or malignancy), and reported good concordance but no information on diagnostic performance [18]. Our results corroborate these previous data and encourage the use of these techniques in the specific population of immunocompromised patients. However, it must be emphasized that certain recognised pathogenic organisms are not included in the panel, and that there are FP whose significance is still unclear.

One striking finding was the contribution and impact of this rapid technique in the antibiotic management of our patients. The median time from sampling to receipt of mPCR results ranged from 2 h to 30 min to 4 h, and antibiotic therapy was adapted early in one case out of five, with modification deemed appropriate. It has been shown that PCR allowed antibiotic de-escalation in 2/3 of cases ruling out mostly MRSA and/or *P.aeruginosa*, and broadening of antibiotic spectrum in 10% of cases [14]. The use of mPCR to diagnose community-acquired and nosocomial pneumonia in critically ill patients has in fact reduced turnaround times [19, 20]. Also, the prospective study by *Guillotin et al.* [21] showed that mPCR reduce the number of broad-spectrum antibiotic days in VAP without increasing therapeutic failure. *Rhee and colleagues* [10], have shown that unnecessary exposure to broad-spectrum antibiotics is associated with an increased mortality. Such has not been confirmed, however, by a recent multicentric randomized controlled trial shows no reduction in days of antibiotic therapy using with mPCR and procalcitonin [22].

The present study has several limitations. Firstly, the study is limited by its retrospective and monocentric nature. Most of our patients (70%) suffered from onco-hematological diseases, making it difficult to extrapolate our results to all types of immunosuppression. Secondly, different sample types were used - tracheal aspirate, miniBAL and BAL - and no cut-off values were used due to lack of validation. Prospective studies are needed to determine the optimal cut-off value in copies/ml for diagnosing infection in this population. Nevertheless, more than 2/3 of the samples (75%) came from BAL. Thirdly, no screening samples were taken during the stay in the intensive care unit. Colonisation, which could play a role in the current infection, could not be specifically analysed in this study. Fourthlly, the therapeutic impact of BFPP mPCR was somewhat difficult to assess due to the retrospective nature of the study. Finally, our study took place during a specific period, the COVID pandemic, which could have altered the local bacterial ecology

## Conclusion

In immunocompromised critically ill patients with ARF requiring invasive ventilation, the BFPP mPCR showed a fair diagnostic performance with a reduced response time. Its NPV allowed us to rule out any infection likely to be induced by any germ of the panel. It can be used to hasten the alteration of the antibiotic spectrum to narrow down or broaden it if MRSA, ESBL/carbapenemase-producing Enterobacteriaceae or non-fermenting gram-negative bacillus bacilli are detected. Nevertheless, further studies are needed to clarify these diagnostic performances and prospectively explore their therapeutic impact.

## Electronic supplementary material

Supplementary Material 1

## Data Availability

No datasets were generated or analysed during the current study.

## Abbreviations

ARF: Acute respiratory failure
BAL: Bronchoalveolar lavage
BFPP: Biofire® filmarray® pneumonia panel
CC: Conventional culture
DNA: Deoxyribonucleic acid
ESBL: Extended-spectrum beta-lactamase
FN: False negative
FP: False positive
ICU: Intensive care unit
Mini-BAL: Mini-bronchoalveolar lavage
mPCR: Multiplex polymerase chain reaction
MRSA: Methicillin resistant* Staphylococcus aureus*
NPV: Negative predictive value
PCR: Polymerase chain reaction
PPV: Positive predictive value
Se: Sensibility
Sp: Specificity
TN: True negative
TP: True positive
VAP: Ventilator associated pneumonia
