# Supplementary material for: Diagnostic performance of Pneumonia multiplex PCR in critically ill immunocompromised patients
Source: Crit Care. 2025 Jul 17;29:310. doi: 10.1186/s13054-025-05528-y (PMC12272964; doi:10.1186/s13054-025-05528-y)
Supplement: Supplementary file 1 — Supplementary Material 1 [file 13054_2025_5528_MOESM1_ESM.docx]

| **Bacteria** | **Resistance** | **Virus** |
| --- | --- | --- |
| *Acinetobacter calcoaceticus-baumannii complex* | CTX-M | *Adenovirus* |
| *Chlamydophila pneumoniae* | IMP | *Coronavirus* |
| *Enterobacter aerogenes* | KPC | *Human Metapneumovirus* |
| *Enterobacter cloacae complex* | NDM | *Human Rhinovirus/Enterovirus* |
| *Escherichia coli* | OXA-48-like | *Influenza A* |
| *Haemophilus influenzae* | SARM (mecA/C + jonction) | *Influenza B* |
| *Klebsiella oxytoca* | VIM | *MERS-CoV* |
| *Klebsiella pneumoniae group* |  | *Parainfluenza Virus 1* |
| *Legionella pneumoniae* |  | *Respiratory Syncytial Virus* |
| *Moraxella catarrhalis* |  |  |
| *Mycoplasma pneumoniae* |  |  |
| *Proteus spp.* |  |  |
| *Pseudomonas aeruginosa* |  |  |
| *Serratia marcescens* |  |  |
| *Staphylococcus aureus* |  |  |
| *Streptococcus* *agalactiae* |  |  |
| *Streptococcus pneumoniae* |  |  |
| *Streptococcus pyogenes* |  |  |

**Table 1.** Multiplex polymerase chain reaction biofire® filmarray® pnuemonia panel of biomérieux®

| 1 | AMOXICILLINE, OXACILLINE, PIPERACILLINE |
| --- | --- |
| 2 | CEFAZOLINE |
| 3 | AMOXCILLINE-ACIDE CLAVULANIQUE, CEFOTAXIME, CEFTRIAXONE |
| 4 | CEFTAZIDIME |
| 5 | PIPERACILLINE-TAZOBACTAM, CEFEPIME |
| 6 | MEROPENEME, IMIPENEME |
| 7 | CEFTAZIDIME-AVIBACTAM, CEFTOLOZANE-TAZOBACTAM, IMIPENEME-RELEBACTAM |
| 8 | CEFIDEROCOL |

**Table 2.** Antibiotics classified in ascending orders of spectrum

| **Variable** | **Population (n = 114)** |
| --- | --- |
| Time between ICU admission and sampling, mean ± SD, in day | 1.2 ± 1.9 |
| Time between intubation and sampling, mean ± SD, in day | 0.3 ± 0.7 |
| Sample modality, n (%) |  |
| *Tracheal aspiration* | 6 (5.3) |
| *Broncho-alveolar lavage* | 85 (74.6) |
| *Mini-broncho-alveolar lavage* | 23 (20.2) |
| Samples treated by BFPP mPCR |  |
| *Positive sample for bacteria testing, n (%)* | 33 (28,9) |
| *Positive sample for virus testing, n (%)* | 10 (8.8) |
| *Detection of resistance gene, n (%)* | 1 (0.8) |
| Sample treated by conventional culture  *Positive sample for bacteria, n (%)*  *Positive sample for bacteria detectable by BFPP mPCR, n (%)* | 46 (40.4)  19 (16,7) |
| *Detection of multi-resistant germs, n (%)* | 1 (0.9) |
| Concordance of bacterial detection for the 2 techniques, n (%) | 96 (84.2) |
| Concordance of resistant germs for both techniques, n (%) | 114 (100.0) |

**Table 3.** Type of samples, microbiological characteristic and result by both techniques: biofire® filmarray® pneumonia panel multiplex polymerase chain reaction and conventional culture

*BFPP: BioFire® FilmArray® Pneumonia Panel; ICU: intensive care unit; mPCR: multiplex Polymerase Chain Reaction; SD: standard deviation*
